# Influence of catheter thickness on respiratory physiology during less invasive surfactant administration in extremely preterm infants

**DOI:** 10.3389/fped.2024.1352784

**Published:** 2024-09-17

**Authors:** Chamindu C. Gunatilaka, Qiwei Xiao, Alister J. Bates, Axel R. Franz, Christian F. Poets, Christian A. Maiwald

**Affiliations:** ^1^ Center for Pulmonary Imaging Research, Cincinnati Children’s Hospital Medical Center, Cincinnati, OH, United States; ^2^ Division of Pulmonary Medicine, Cincinnati Children’s Hospital Medical Center, Cincinnati, OH, United States; ^3^ Department of Pediatrics, University of Cincinnati, Cincinnati, OH, United States; ^4^ Department of Neonatology, University Children’s Hospital Tübingen, Tübingen, Germany; ^5^ Center for Pediatric Clinical Studies (CPCS), University Hospital Tübingen, Tübingen, Germany

**Keywords:** less invasive surfactant administration (LISA), minimal invasive surfactant therapy (MIST), respiratory distress syndrome, ELGANS, respiratory physiology

## Abstract

**Introduction:**

Delivering surfactant via thin catheters (minimal-invasive surfactant therapy (MIST); less invasive surfactant administration (LISA)) has become a common procedure. However, the effect of tracheal obstruction caused by catheters of different sizes on tracheal resistance in extremely low gestational age newborns (ELGANs) is unknown.

**Methods:**

To investigate the effect of catheters size 3.5, 5 and 6 French on airway resistance in ELGANs of 23–28 weeks gestational age during LISA, we performed calculations based on Hagen-Poiseuille's law and compared these with a clinically and physically more accurate method: computational fluid dynamics (CFD) simulations of respiratory airflow, performed in 3D virtual airway models derived from MRI.

**Results:**

The presence of the above catheters decreased the cross-sectional area of the infants' tracheal entrance (the cricoid ring) by 13–53%. Hagen-Poiseuille's law predicted an increase in resistance by 1.5–4.5 times and 1.3–2.6 times in ELGANs born at 23 and 28 weeks, respectively. However, CFD simulations demonstrated an even higher increase in resistance of 3.4–85.1 and 1.1–3.5 times, respectively. The higher calculated resistances were due to the extremely narrow remaining lumen at the glottis and cricoid with the catheter inserted, resulting in a stronger glottal jet and turbulent airflow, which was not predicted by Hagen-Poiseuille.

**Conclusion:**

Catheter thickness can greatly increase tracheal resistance during LISA-procedures in ELGANs. Based on these models, it is recommended to use the thinnest catheter possible during LISA in ELGANs to avoid unnecessary increases in airway resistance in infants already experiencing dyspnea due to respiratory distress syndrome.

## Introduction

Surfactant therapy via less invasive surfactant administration (LISA) or minimally-invasive surfactant therapy (MIST) in neonates is increasingly used and its introduction into routine care in neonatal intensive care units in Europe occurred quite rapidly (1–4).

However, there is also critique concerning this method (5, 6). Compared to more invasive methods, the distribution of surfactant seems worse (7) and there is a higher risk of failure particularly in extremely low gestational age neonates (ELGANs) (8). A potential reason for this could be an impaired lung ventilation during LISA. In their critique of the LISA-method, De Luca et al. also referred to the influence of catheter thickness and argued that this can be calculated by using Hagen-Pouiselle's law (5). However, because Hagen-Pouiselle's law requires a circular cross-sectional area and a laminar flow and both conditions are not fulfilled during LISA, we performed a more detailed investigation on this topic.

In Germany, various approved and specifically designed LISA/MIST-catheters (e.g., Neofact®—Lyomark Pharma, Oberhaching, Germany and Surfcath™—Vygon, Aachen, Germany) and non-approved catheters intended for other purposes (e.g., gastric tubes, umbilical vein catheters, etc.) are being used for LISA (9, 10). While outer diameters of the CE-marked, approved catheters are 3.5 (1.2 mm; Neofact®) or 6 French (2.0 mm; Surfcath™) respectively, non-approved catheters reportedly vary between 3.5 and 5 French (1.2 and 1.7 mm).

The narrowest part of the airway is the cricoid ring which has an inner diameter that varies between 2.7–2.8 mm in infants with a gestational age (GA) of 23 weeks and 3.2–3.3 mm in infants with a GA of 28 weeks, respectively (11, 12).

This is not much greater than catheter thickness and could therefore represent a relevant obstacle to the infants’ spontaneous respiration, since tracheal obstruction by the catheter will likely increase airway resistance, resulting in an increased work of breathing (WOB), decreased tidal volume (Vt) and minute ventilation (MV). Considering the law of Hagen-Poiseuille, a reduction of the inner diameter of the airway increases resistance by the fourth power (13) *“or the fifth power, in the case of non-laminar flow” … “because of the non-perfectly circular shape”* of the tracheal cross-sectional area (5). It can therefore be assumed that the airway obstruction induced by the catheter during LISA/MIST will have the greatest impact on breathing in the smallest infants. And it may also explain why GA <28 weeks is an independent risk factor for LISA-failure (8).

However, in addition to the obstruction itself, the neonatal airway has some unique features that makes it much more difficult for the infant to compensate such a loss in Vt.

(1) WOB in preterm infants is mainly done by the diaphragm (14). However, stronger contractions of the diaphragm (to overcome increased resistance) would be partially off-set by the high elasticity of the chest wall (15).
(2) The horizontal position of the ribs hardly allows any support from thoracic muscles (14).
(3) The respiratory muscles mainly consist of quickly exhaustible type I muscle fibers (16).
(4) Pharynx, larynx and trachea are softer than in older infants and tend to collapse more quickly (17), which additionally raises resistance. This must be particularly true, when the flow velocity will be raised to maintain Vt/MV during an obstruction.

One should also consider the altered postnatal adaptation of ELGANs, where lung fluid clearance of the airways by aquaporin-4 channels (AQP4) is less effective (18), and the additional impact of the respiratory distress syndrome (RDS), which is the reason these infants should be treated via LISA/MIST. RDS decreases the functional residual capacity (19) and accelerates respiratory rate (20). The latter factor limits the ability to compensate a loss of Vt by the catheter-obstruction.

The points discussed above triggered this investigation into the potential influence of catheter thickness during LISA/MIST on respiratory physiology, as we suspected that the use of thicker catheters could affect spontaneous breathing during LISA (and thus possibly also the homogeneous distribution of surfactant), particularly in ELGANs, and could therefore represent a potential risk factor for treatment failure.

## Material and methods

### Method selection

We calculated the catheter-induced change in airway resistance using two methods. First, we calculated the pressure drop (and therefore resistance) of a laminar flow within a tube using Hagen-Poiseuille's law to estimate changes in the airway resistance, as discussed by specialists in this field (5). The rationale for using Hagen-Poiseuille's law is that it is a relatively simple calculation and can thus be performed quickly for any combination of patient and catheter size. However, the theory behind this law makes several assumptions about flow and geometry (listed below) which may not apply to tracheal airflow. Therefore, computational fluid dynamics (CFD) simulations, which are not based on the same assumptions, and are therefore capable of producing more accurate results, were also performed. The downside to CFD simulations is that they are computationally expensive to perform, and patient-specific simulations require medical imaging of the airway to create its geometry.

### Theoretical calculations

The Hagen-Poiseuille model of airway resistance was made by first calculating the cross-sectional area (CSA) of the associated tracheal lumen $\left(\mathrm{CS}A_{\mathrm{lumen}}\right)$ at the narrowest part (cricoid ring) in infants with an GA of 23–28 weeks [diameters were calculated according to (11, 12)]. The CSA of the catheters were also calculated using 3.5, 5, and 6 French tubes $\left(\mathrm{CS}A_{\mathrm{catheter}}\right)$. For each combination of patient and catheter size, the remaining CSA of the lumen with the catheter in place was calculated $\left(\mathrm{CS}A_{\mathrm{lumen}}-\mathrm{CS}A_{\mathrm{catheter}}\right)$, and the radius of a circle with this remaining area $\left(r_{\mathrm{remaining}}\right)$ calculated as follows:

$$r_{\mathrm{remaining}}=\sqrt{\frac{\mathrm{CS}A_{\mathrm{lumen}}-\mathrm{CS}A_{\mathrm{catheter}}}{\pi }}$$

After that, the percentage of the remaining radius ($r_{\mathrm{ratio}}=r_{\mathrm{remaining}}/r_{\mathrm{trachea}}\;$, where $r_{\mathrm{trachea}}$ is the radius of the trachea) was calculated to determine the increase in resistance (ΔRes) by application of Hagen-Poiseuille's law $\left(\Delta \mathrm{Res}=1/(r_{\mathrm{ratio}}^{4})\right)$. This law estimates the resistance along a tube due to flow based on the following assumptions: laminar flow; constant circular CSA; no flow acceleration; incompressible liquid; diameter much smaller than the length of the tube. Since several of these assumptions are not true for respiratory airflow, a second method was also used to calculate the change in resistance (21).

All calculations were performed using Microsoft Excel 2010.

### Computational fluid dynamics (CFD)

A neonatal intensive care unit patient at a post-menstrual age of 40 weeks was enrolled with approval from the Institutional Review Board and parental consent. The patient had no known airway or lung disease, was considered as a control for the purpose of this study and was imaged using ultrashort echo time magnetic resonance imaging (MRI) technique. Next, a three-dimensional airway model was created via segmentation of the MR images as previously described (22–29). To create airway models at sizes equivalent to different ages, the three dimensional airway model of the 40 week old subject was downscaled uniformly until an average tracheal diameter of 3.0 and 3.8 mm was reached, which was equivalent to be the average diameter of an infant with a GA of 23 weeks and 28 weeks, respectively (11, 12). Breathing rate was set to 80 breaths/minute and inspiration time to 1/3 of a respiratory cycle. Tidal volume was calculated as 5.75 ml/kg bodyweight based on weights of 600 g for the 23-week model and 1,050 g for the 28-week model [50th percentile of female infants with 23 and 28 weeks of gestation (30)]. PEEP was set to 6 cmH_2_O, as this is the standard procedure at the neonatal intensive care unit in Tuebingen. CFD simulations were performed using Simcenter STAR-CCM+ 14.04.011-R8 (Siemens Digital Industries Software) commercial software package. These respiratory airflow simulations have been validated previously (31). CFD simulations model the airflow for a single respiratory cycle without catheters and subsequently with nasally guided catheters of 3.5, 5, and 6 French diameters were inserted 1.5 cm below the vocal cords into the trachea of the infant with a GA of 23 weeks and 2.0 cm in the infant with a GA of 28 weeks (32). For each simulation, airway resistance in the trachea at peak inspiration and the total pressure drop between the nostrils and the end of the main bronchi at peak expiration were calculated.

## Results

### Theoretical calculations

**Figure 1:**
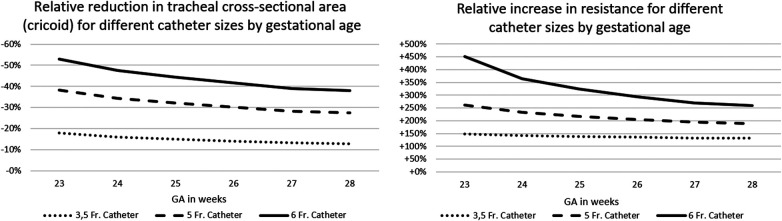
Overview of theoretical calculations based on the Law of Hagen–Pouiselle.

Depending on the diameter of the LISA-catheter and CSA of the trachea, the “remaining CSA” was reduced by 13%–53%. This resulted in a theoretical increase in resistance by a factor of ×1.3–4.5. The relevance of the differences increased exponentially with immaturity (i.e., lower tracheal CSA) of the infant. For details, see Figure 1 and calculations, see Table 1.

**Table 1:** Calculations for the use of catheters of different thickness during LISA, based on Hagen–Pouiselle's law.

	Infant's		3.5 Fr. Catheter					
GA	Diameter of cricoid ring (*d_t_*) in mm	Tracheal cross sectional area in mm^2^	Catheter diameter (*d_c_*) in mm	Catheter cross sectional area in mm^2^	ΔObstr (reduction of the cross sectional area)	Radius resulting trachea in mm	Percentage of resulting radius	Raising of resistance
23	2.75	5.9	1.16	1.1	−18%	1.25	90.7%	×1.5
24	2.9	6.6	1.16	1.1	−16%	1.33	91.7%	×1.4
25	3	7.1	1.16	1.1	−15%	1.38	92.2%	×1.4
26	3.1	7.5	1.16	1.1	−14%	1.44	92.7%	×1.4
27	3.2	8.0	1.16	1.1	−13%	1.49	93.2%	×1.3
28	3.25	8.3	1.16	1.1	−13%	1.52	93.4%	×1.3

	Infant's		5 Fr. Catheter					
GA	Diameter of cricoid ring (*d_t_*) in mm	Tracheal cross sectional area in mm^2^	Catheter diameter (*d_c_*) in mm	Catheter cross sectional area in mm^2^	ΔObstr (reduction of the cross sectional area)	Radius resulting trachea in mm	Percentage of resulting radius	Raising of resistance
23	2.75	5.9	1.7	2.3	−38%	1.1	78.6%	×2.6
24	2.9	6.6	1.7	2.3	−34%	1.2	81.0%	×2.3
25	3	7.1	1.7	2.3	−32%	1.2	82.4%	×2.2
26	3.1	7.5	1.7	2.3	−30%	1.3	83.6%	×2.0
27	3.2	8.0	1.7	2.3	−28%	1.4	84.7%	×1.9
28	3.25	8.3	1.7	2.3	−27%	1.4	85.2%	×1.9

	Infant's		6 Fr. Catheter					
GA	Diameter of cricoid ring (*d_t_*) in mm	Tracheal cross sectional area in mm^2^	Catheter diameter (*d_c_*) in mm	Catheter cross sectional area in mm^2^	ΔObstr (reduction of the cross sectional area)	Radius resulting trachea in mm	Percentage of resulting radius	Raising of resistance
23	2.75	5.9	2	3.1	−53%	0.9	68.6%	×4.5
24	2.9	6.6	2	3.1	−48%	1.1	72.4%	×3.6
25	3	7.1	2	3.1	−44%	1.1	74.5%	×3.2
26	3.1	7.5	2	3.1	−42%	1.2	76.4%	×2.9
27	3.2	8.0	2	3.1	−39%	1.2	78.1%	×2.7
28	3.25	8.3	2	3.1	−38%	1.3	78.8%	×2.6

### CFD

**Figure 2:**
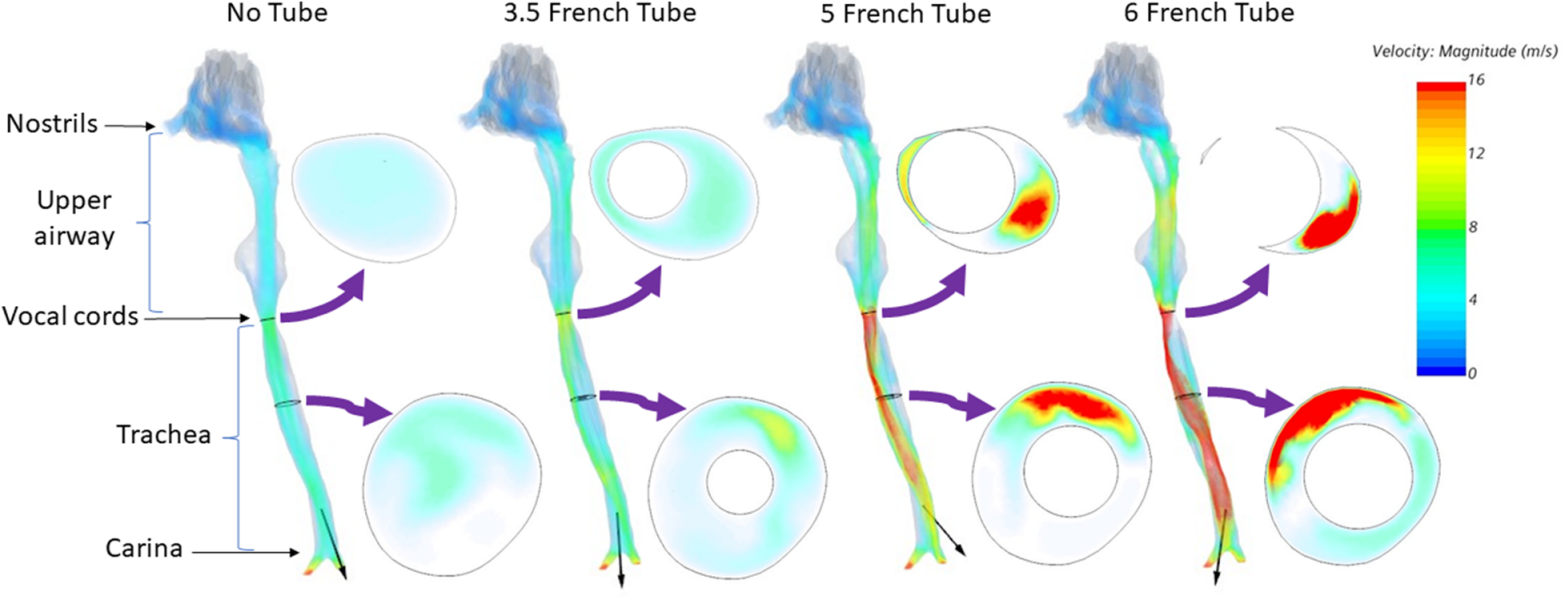
Airflow velocity at peak inspiration for an infant of 23 weeks’ gestation. Left to right: No tube, 3.5 French tube, 5 French tube, and 6 French tube. The black arrow on each airway represents the airflow direction.

CFD simulations demonstrated a clear increase in airflow velocity at peak inspiration in the infant's trachea as the catheter increased in size (Figure 2). There was a high velocity jet from the vocal cords into the trachea when a 6 French catheter was inserted into the airway. However, the increase in airflow velocity was minimal when a 3.5 French catheter was inserted, although the airflow pattern was also changed. When 5 French and 6 French catheters were placed, airflow was shifted to the anterior aspect of the tracheal lumen, with little flow in the posterior part of the airway.

**Figure 3:**
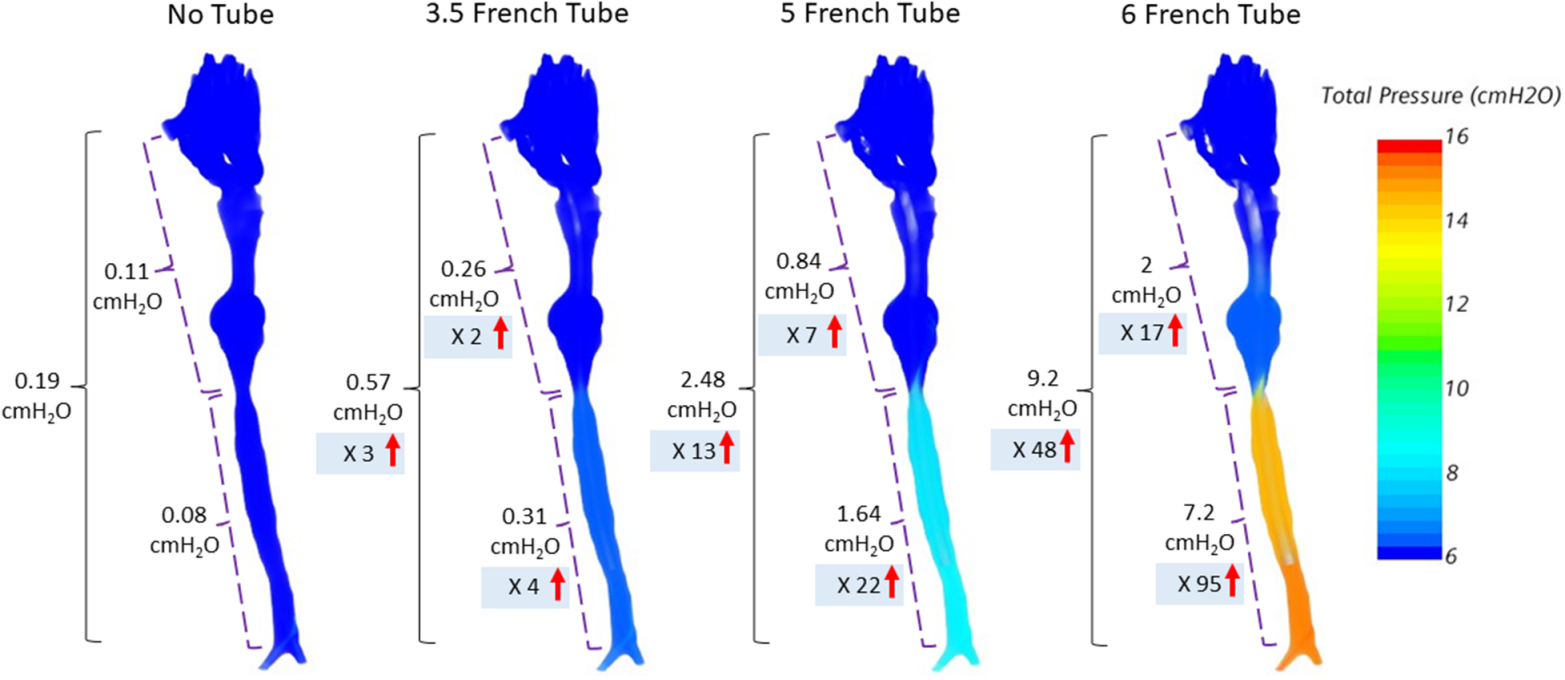
Total pressure drop between nostrils and carina at peak expiration for an infant at a GA of 23 weeks. The total pressure drop between nostrils to carina is further divided into upper airway and trachea. The insertion of a 6 French catheter into the airway results in a 48-times increase in total pressure drop between the nostrils and carina compared to the unobstructed airway without catheter.

We then calculated the pressure drop between nostrils and the carina at peak expiration expected from the increase in airflow resistance due to catheter obstruction. Figure 3 demonstrates the total pressure drop in a preterm infant of 23 weeks gestation.

**Figure 4:**
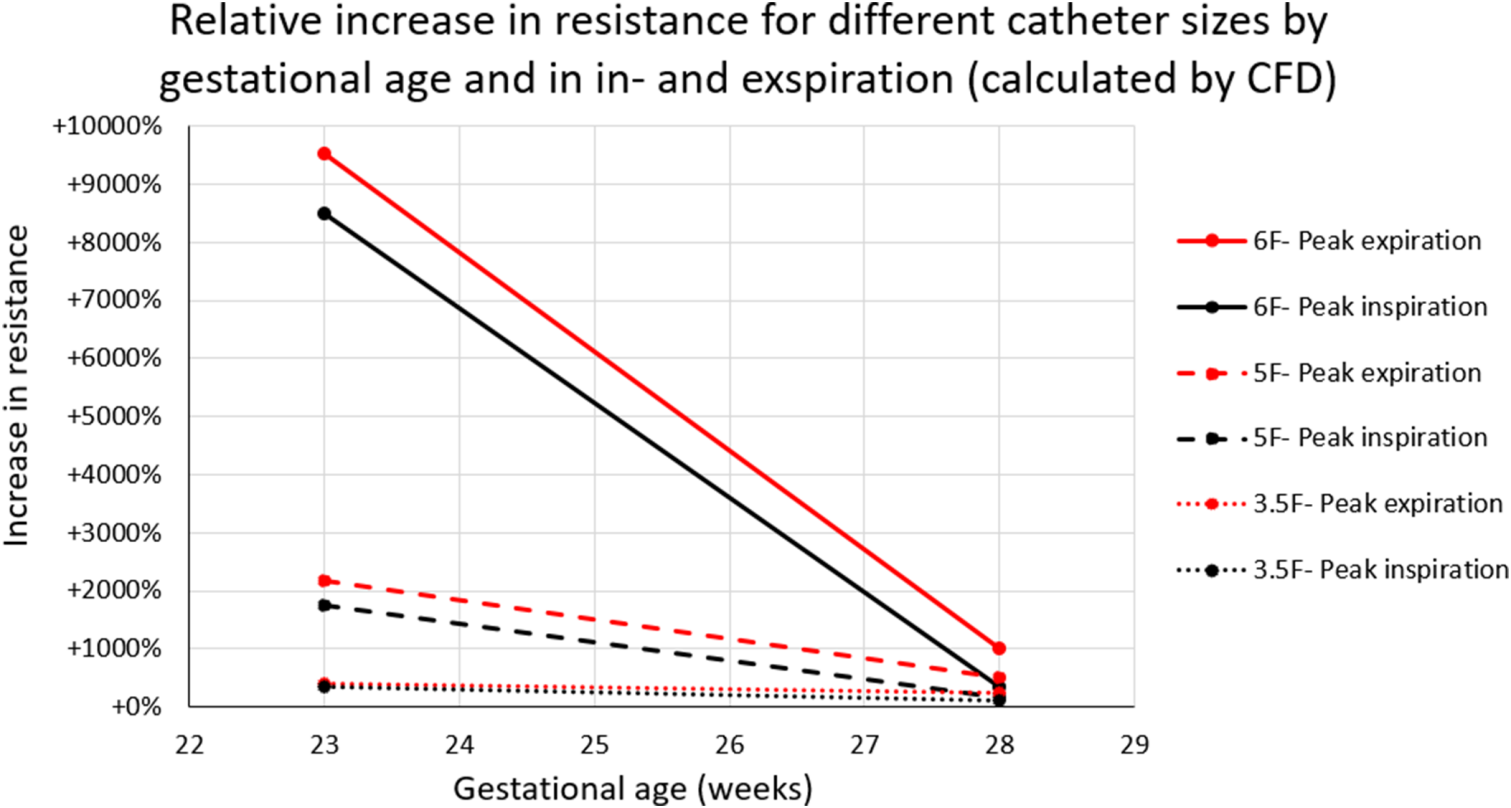
Increase in tracheal resistance at peak inspiration (black) and peak expiration (red) caused by a tracheal catheter in infants at 23- and 28-weeks’ GA. Tracheal resistance was raised by more than 85 times when a 6F catheter was inserted into the airway of an infant at 23 weeks’ GA.

The tracheal resistance at peak in- and expiration significantly increased with immaturity and thickness of the tracheal catheter. Simulations in an infant at 23 weeks’ gestation demonstrated a threefold (3.5 Fr.), 17-fold (5 Fr.), and 85-fold (6 Fr.), respectively, increase in airway resistance at peak inspiration (Figure 4).

## Discussion

The clinical relevance of our findings arises from the purely logical consideration that in a critically ill infant with respiratory distress undergoing LISA, any additional, procedure-related WOB should be reduced to a minimum. The reported calculations serve to illustrate the potential magnitude of catheter-related factors during LISA.

Although clinical trials have shown the benefit of LISA in ELGANs, the pertinent Cochrane review (33) included only limited data on extremely preterm infants. Only Kribs et al. (34) reported a subgroup analysis of infants at 23–24 weeks gestation and found no benefit for LISA. Additionally, GA <28 weeks is an independent risk factor for LISA-failure (8) and surfactant distribution was shown to be worse in LISA compared to invasive surfactant application methods in lambs (7). Therefore, an improvement of the ventilatory situation during LISA (e.g., by a reduction in catheter size) might be helpful in these infants.

In-silico calculations were chosen for this study since *in vivo* measurements of airway resistance in spontaneously breathing infants on non-invasive respiratory support during LISA procedures are impractical. However, measurements of flow or electrical impedance tomography (35) might help to better understand these physiological aspects in the future.

Hagen-Poiseuille's law assumes a laminar flow in an ideal tube. Such theoretical assumptions are common in physical calculations (36) and were also mentioned in relation to LISA (5), but only rarely reflect clinical reality: as shown, CFD airflow becomes turbulent due to an increase in airflow velocity caused by catheter-related airway obstruction, and the resulting crescent-shaped CSA increases airway resistance substantially. Even assuming a fifth power relationship as suggested by De Luca et al. (corresponding to a 1.6–6.6-fold increase), one would strongly underestimate the increase in resistance.

Due to the above-mentioned features of the neonatal airway, ELGANs only have two options to compensate catheter-mediated airway obstruction. They can increase their respiratory rate or the pressure gradient (*Δ*P) across the trachea, which would have to be accomplished by an increase in diaphragmatic contractility. However, a higher velocity of tracheal gas flow leads to a reduced distending pressure according to the Bernoullie principle during inspiration and thus to an increased risk of airway collapse, a further increase in resistance and reduced effectiveness of the applied WOB. Taken together, the capacity for compensation of such a catheter-related partial airway obstruction appears to be limited in ELGANs suffering from RDS.

The role of PEEP during LISA with respect to airway resistance, effective Vt and WOB appears unpredictable with our model. On one hand, partial airway obstruction, particularly if combined with an increased respiratory rate, may lead to inadvertent PEEP, which might increase dead-space and therefore additionally reduces effective tidal volume. On the other hand, PEEP will be near zero during catheter insertion (due to an open mouth during laryngoscopy). Therefore, it remains unclear whether the catheter-related obstruction prevents any build-up of PEEP or will lead to an auto-PEEP.

Limitations of this study include that it relies on in-silico simulations and theoretical calculations based on physical principles rather than *in vivo* measurements. The calculations and simulations do not consider potential partial compensation by increased breathing efforts of the infant and the role of PEEP. In addition, our CFD calculations were based on a term infant whose proportions were uniformly scaled down to represent a premature infant. The anatomical proportions may vary in reality and therefore it is debatable whether the CFD results of one MRI can be considered representative of the target population.

However, in summary, we conclude that the use of thicker catheters in ELGANs may lead to decreased tidal volume due to increased resistance during the LISA procedure, potentially also affecting surfactant distribution (7, 37), and therefore it seems to be possible, that the use of thicker catheters increase failure rates of LISA in ELGANs.

## Conclusion

The present calculations show that thickness of the selected catheter for LISA procedures matters, particularly in the most immature ELGANs with a small trachea. The increase in the infant's tracheal resistance and WOB can be relevant and may contribute to LISA failures. These findings may be relevant to the clinical setting, despite our results being only based on in silico modeling and theoretical considerations rather than *in vivo* measurements. We recommend that catheters selected for LISA in ELGANs should be as thin as possible—but the disadvantage of a potentially more difficult LISA procedure with a thinner and hence more flexible catheter have to be considered.

## Data Availability

The original contributions presented in the study are included in the article/Supplementary Material, further inquiries can be directed to the corresponding author.
